# Psychometric evaluation and refinement of the 7DHW questionnaire for the German population

**DOI:** 10.3389/fpsyg.2026.1812409

**Published:** 2026-06-15

**Authors:** Inês Santos Silva, Frank Schifferdecker-Hoch, Michael Hollmann, Luísa Soares

**Affiliations:** 1 2256c.health Unipessoal LDA, Lisbon, Portugal; 2 FPZ GmbH, Cologne, Germany; 3 Departamento de Psicologia, Faculdade de Artes e Humanidades, Universidade da Madeira, Funchal, Portugal

**Keywords:** health knowledge, holistic wellbeing, mental and physical health, psychometrics, questionnaire validation

## Abstract

**Background:**

Holistic conceptualizations of health emphasize the integration of physical, psychological, social, occupational, and environmental dimensions. However, psychometrically robust instruments that operationalize such multidimensional frameworks remain limited. This study aimed to examine the internal structure, reliability, and construct validity of the Holistic Scale of Body and Mental Health and Wellbeing, based on the 7 Dimensions of Holistic Wellbeing (7DHW) model, in a German adult sample.

**Methods:**

A cross-sectional study was conducted with 677 adults in Germany (age range 22–86 years). Participants completed the 7DHW questionnaire online. Psychometric evaluation included exploratory and confirmatory factor analyses (EFA and CFA) to assess dimensionality, internal consistency analyses using Cronbach's α and McDonald's ω, and known-groups comparisons across gender and age. Item-level diagnostics were conducted to evaluate scoring and conceptual coherence, particularly for poorly performing sub-scales.

**Results:**

The findings provided partial support for the conceptual framework while revealing substantial multidimensionality within several domains. Dimensions A (Self-Esteem), C (Social Relationships), D (Environment), and G (Sense of the Future) demonstrated strong internal consistency, and Dimension E (Meaningful Work) showed acceptable reliability. The original Body Image (B) and Health Knowledge and Behavior (F) dimensions showed inadequate psychometric performance. Following item-level diagnostics, Dimension B was refined through reverse coding and item reduction, resulting in acceptable reliability, although its factorial structure remains provisional. Dimension F demonstrated a heterogeneous structure and was better represented as a set of exploratory sub-domains (e.g., preventive behavior, dietary behavior), with variable reliability across components. Known-groups analyses revealed expected differences by gender and age across selected dimensions and sub-domains, supporting construct validity.

**Conclusions:**

The results indicate that several domains are inherently multidimensional and may be better conceptualized as clusters of related subcomponents rather than strictly unidimensional scales. Overall, the instrument demonstrates promising psychometric properties at the domain level, while highlighting the need for further refinement and independent validation of specific dimensions.

## 1 Introduction

The concept of health and wellbeing has evolved significantly over the past decades, transitioning from a reductionist biomedical model to a holistic understanding that integrates physical, psychological, social, and environmental factors (Engel, 1977; Kickbusch, 1989; McLeroy et al., 1988). The World Health Organization (WHO) defines health as “a state of complete physical, mental, and social wellbeing and not merely the absence of disease” (Organization, 1946), a definition that has inspired a shift in research and practice toward multidimensional models of wellbeing (Ryff, 1995; Seligman, 2011; Keyes, 2002). Several studies in the field of psychology have explored the more holistic and comprehensive aspects of the concept of wellbeing. These models aim to reflect the complexity of the human experience and capture interdependent domains that contribute to the overall quality of life (Dodge et al., 2012; Ryff and Singer, 1998; Diener et al., 1999). This approach is also aligned with recent studies on emotional and social wellbeing in stroke survivors, which emphasize the role of digital interventions in understanding and enhancing wellbeing in complex health contexts (Santos Silva et al., 2025, 2024; Rodrigues et al., 2025). While several theoretical and empirical models have emerged—such as Ryff's psychological wellbeing dimensions and Keyes' flourishing framework (Ryff, 1995; Keyes, 1998)—many existing instruments tend to focus on isolated aspects of wellbeing or lack cultural and contextual adaptability (Huppert and So, 2013; Hicks et al., 2013). As highlighted in recent systematic reviews (Diener and Chan, 2011; Bai and Lazenby, 2015), there is a growing demand for validated, multidimensional, and flexible tools that can be applied in diverse health, occupational, and community settings. In particular, tools that encompass both body and mental health under a single framework remain limited (Brown and Applegate, 2012; Kaveh et al., 2016). Especially in populations with chronic conditions such as stroke, where emotional and social dimensions of recovery are often overlooked (Silva et al., 2020; Santos Silva et al., 2024; Rodrigues et al., 2025).

To address this gap, the 7 Dimensions of Holistic Wellbeing (7DHW) model was developed, grounded in the WHO's core principles of holistic health (dos Santos Silva et al., 2024). This model identifies seven foundational dimensions that collectively define holistic wellbeing: **(A) stable self-esteem** (Rosenberg, 1965b; Orth et al., 2012), **(B) positive body image** (Cash and Smolak, 2011; Tiggemann, 2004), **(C) supportive social relationships** (Cohen and Wills, 1985; Kawachi and Berkman, 2001), **(D) an intact and safe environment** (Evans, 2003; Frumkin, 2001), **(E) meaningful work in healthy conditions** (Wrzesniewski et al., 1997; Steger et al., 2012), **(F) access to health knowledge and care** (Nutbeam, 2000; Kickbusch et al., 2013), and **(G) a present and future worth living** (Seligman, 2011; Snyder et al., 2002). These dimensions are conceived as interconnected, each influencing the others, and together forming a coherent framework to evaluate the full spectrum of individual wellbeing.

Building on this theoretical foundation, we developed the ***Holistic Scale of Body and*** ***Mental Health and Wellbeing***. This Holist Scale is a psychometric instrument designed to assess these seven domains through self-report. A pilot study previously explored the test–retest consistency of the questionnaire in a Portuguese sample, offering promising preliminary insights into its structure and applicability (dos Santos Silva et al., 2024).

The current study represents a further and essential step in the validation process of the **7DHW questionnaire in German population**, using a large-scale German adult sample (*N* = 677). This sample spans a broad demographic range (age 22–86), with representation from diverse professional and educational backgrounds, and includes individuals with and without mental or physical health conditions. Through rigorous statistical procedures—including exploratory and confirmatory factor analysis, internal consistency checks, and validity assessments—this study aims to evaluate the reliability and psychometric robustness of the instrument (Paiva et al., 2014; Orth and Robins, 2014).

The purposes of this study are threefold:

- To examine the internal structure of the 7DHW questionnaire and confirm whether the seven theorized dimensions are empirically supported.
- To assess the internal consistency and reliability of each dimension across the sample (Cronbach, 1951; Ravinder and Saraswathi, 2020).
- To establish preliminary evidence for construct validity, including associations between the wellbeing dimensions and health-related variables.

In doing so, we contribute to the growing field of holistic health measurement by filling the gaps of the existing questionnaire. We introduce and evaluate an evidence-based, theory-driven instrument that holds potential for application in clinical, occupational, educational, and public health contexts (Huppert and So, 2013; Hicks et al., 2013; Baras et al., 2018).

## 2 Background

In recent years, the concept of wellbeing has garnered increasing attention across various disciplines, including psychology, medicine, public health, education, and organizational sciences (Seligman, 2011; Dodge et al., 2012; Breslow, 1999). This interest has extended to psycho-oncology and stroke recovery, where emotional wellbeing is increasingly recognized as a key factor in long-term outcomes (Paiva et al., 2023; Soares et al., 2024; Silva et al., 2020; Santos Silva et al., 2025; Rodrigues et al., 2025; Santos Silva et al., 2024). This multidimensional construct extends beyond the traditional notion of health as merely the absence of disease. As defined by the *World Health Organization* (WHO) (Organization, 1946), health is “a state of complete physical, mental and social wellbeing,” which underscores the need for comprehensive frameworks and instruments capable of evaluating wellbeing holistically and across life domains.

### 2.1 Defining wellbeing and wellness

**Wellbeing** and **wellness** are closely linked yet distinct concepts. While wellbeing generally refers to a subjective state of life satisfaction, positive affect, and functioning (Dodge et al., 2012), wellness is often considered an active process of becoming aware of and making choices toward a healthy and fulfilling life (Helne, 2018). Both concepts encompass psychological, physical, emotional, social, occupational, and spiritual dimensions, and they require integrative models for full understanding and application (Hicks et al., 2013; Brown and Applegate, 2012). Research has also stressed the need to incorporate historical perspectives and clinical practice evolution in understanding wellbeing as a lived, contextualized construct (Soares, 2023). Traditional models, such as *Ryff's Psychological Wellbeing* (PWB) (Ryff, 1995) and *Keyes' model of flourishing* (Keyes, 1998), have emphasized autonomy, personal growth, and positive relationships as pillars of psychological health. Other instruments, such as the *Warwick-Edinburgh Mental Wellbeing Scale* (WEMWBS) and the *WHO-5 Wellbeing Index*, focus more narrowly on mental or emotional wellbeing (Tennant et al., 2007; Bech, 2012). However, these models often lack a comprehensive integration of environmental, occupational, or future-oriented dimensions—gaps that recent holistic models attempt to bridge (dos Santos Silva et al., 2024; Rosenberg, 1965a).

### 2.2 Instruments and approaches in wellbeing assessment

A recent systematic literature review highlights a proliferation of wellbeing assessment instruments developed over the past two decades (Hicks et al., 2013; Diener and Chan, 2011). These tools vary in scope, from general mental health surveys to context-specific instruments targeting workplace wellbeing (Bakker and Demerouti, 2007), patient-reported outcomes in healthcare (Velikova et al., 2004), or wellbeing among aging populations and adolescents (Tiggemann, 2004). The review emphasizes the need for psychometrically robust, cross-culturally adaptable tools that reflect the interconnected nature of wellbeing domains (Nutbeam, 2000; Kickbusch et al., 2013). For example, the Comprehensive Wellbeing Scale (CWBS), developed by Sham et al. (2021), integrates intrapersonal and transpersonal wellbeing dimensions and has shown promise for use in populations recovering from mental health issues. Similarly, the Perceived Wellness Survey (PWS) (Kaveh et al., 2016) and instruments developed under the NIOSH Worker Wellbeing initiative have been used to assess organizational wellbeing and occupational health risks (Zwetsloot and Leka, 2010). These efforts confirm the growing relevance of multidimensional wellbeing assessments across clinical, educational, and occupational contexts. Emerging studies also show the potential of messaging platforms, such as WhatsApp, in promoting peer-based emotional support within care communities (Santos Silva et al., 2024; Rodrigues et al., 2025).

### 2.3 A holistic perspective: from theory to measurement

Despite the diversity of existing instruments, a persistent challenge is the development of tools that fully align with the WHO's holistic definition of health, capturing the nuances of both physical and mental health, and addressing the interdependence of personal, social, environmental, and existential factors (Antonovsky, 1987; Lindström and Eriksson, 2006; Antonovsky, 1979). In response, the ***7 Dimensions of Holistic Wellbeing*** ***(7DHW)*** model (dos Santos Silva et al., 2024) was introduced as a theory-driven framework, identifying seven core dimensions: Self-Esteem, Body Image, Social Relationships, Environmental Wellbeing, Meaningful Work, Health Knowledge and Behavior, and Sense of the Future. This model serves as the foundation for the development of the Holistic Scale of Body and Mental Health and Wellbeing, a psychometric tool designed to assess wellbeing in its full complexity. The questionnaire was specifically designed to explore both internal and external domains of wellbeing, recognizing that factors such as self-worth, body perception, connection to others, and hope for the future are deeply intertwined with workplace satisfaction, access to care, and environmental awareness (Evans, 2003; Wrzesniewski et al., 1997; Steger et al., 2012).

### 2.4 Applications and potential use cases

Due to its comprehensive design, this instrument holds significant potential across multiple fields:

- **Organizational wellbeing:** Companies and institutions can use the questionnaire to assess employee wellbeing beyond job satisfaction—capturing psychosocial safety, meaning, and future orientation, which are crucial for retention, engagement, and mental health (Baras et al., 2018; Zwetsloot and Leka, 2010; Burlakova et al., 2020; Roque and Soares, 2011).
- **Rehabilitation and healthcare:** The tool can be used to monitor rehabilitation outcomes among individuals recovering from chronic illness or trauma, providing a more nuanced view of patient recovery beyond physical metrics (Lawrence and Kinn, 2012; Brasier et al., 2016; Stanton et al., 2015).
- **Self-awareness and coaching:** For individuals seeking to reflect on their current state of wellbeing, the questionnaire offers a structured way to identify strengths and vulnerabilities across various life domains, making it suitable for coaching, counseling, or preventive health programs (Brown and Applegate, 2012; Kawachi and Berkman, 2001; Ryff and Singer, 2006).

### 2.5 The need for validation

While the theoretical model has been previously introduced and piloted in small-scale studies, the current research represents a crucial step in establishing the empirical validity of the instrument. Drawing on a large German adult sample, this study aims to evaluate the questionnaire's internal consistency, factorial structure, and construct validity, to provide a reliable tool for cross-contextual and cross-cultural applications (Kaveh et al., 2016; Orth and Robins, 2014; Baras et al., 2018).

## 3 Methods

The methodological approach of this study was designed to ensure a comprehensive validation of the Holistic Scale of Body and Mental Health and Wellbeing. Data were collected from a large adult sample in Germany, encompassing diverse age and gender groups. Analyses were conducted according to established guidelines for psychometric evaluation, including reliability testing, factor analysis, and group differentiation procedures. Statistical analyses were conducted using SPSS (Version 29) and Jamovi, encompassing both exploratory and confirmatory approaches to assess dimensionality, as well as inferential tests to evaluate preliminary construct validity.

### 3.1 Participants

A total of 677 individuals residing in Germany participated in this study. The sample consisted of a diverse adult population ranging in age from 22 to 86 years (M = 53.95, SD = 11.42), with an approximately normal distribution. The gender distribution was predominantly female (*n* = 538, 79.5%), with male participants representing 20.4% (*n* = 138), and one participant identifying as diverse (0.1%). Regarding physical health, 196 participants (28.9%) reported a diagnosed physical condition, most frequently herniated disc, osteoarthritis, scoliosis, and high blood pressure. Around 89 individuals (13.1%) reported mental health issues, such as depression, burnout, or anxiety disorders. Medication usage was noted in 274 participants (40.5%), and psychotherapy experience was reported by 186 (27.5%). The sample also showed diversity in occupational status and education levels. Most participants were professionally active (*n* = 533, 78.7%), while 144 (21.3%) were retired. Education levels ranged from basic secondary schooling to postgraduate degrees, with 143 participants (21.1%) holding a university degree and 16 (2.4%) holding a doctoral or postdoctoral qualification.

### 3.2 Instrument

The instrument applied in this study was the Holistic Scale of Body and Mental Health and Wellbeing, a self-report questionnaire developed to assess wellbeing across seven dimensions derived from the 7 Dimensions of Holistic Wellbeing (7DHW) theoretical model (dos Santos Silva et al., 2024). These seven core dimensions are grounded in the WHO definition of health and include:

1. **Self-esteem:** a stable and positive sense of self-worth and confidence;
2. **Body image:** a positive perception of one's own body;
3. **Social relationships:** quality and depth of supportive social connections;
4. **Environment:** perception of the stability and health of one's surrounding environment;
5. **Meaningful work:** sense of purpose and satisfaction with working conditions;
6. **Health knowledge and behavior:** awareness, access, and engagement in health-related behaviors;
7. **Sense of the future:** hope, purpose, and engagement with present and future life.

Each item was assigned to a dimension based on the theoretical structure of the 7DHW model, which specifies the core components of each domain of holistic wellbeing. Items in Dimension A capture emotional self-regard, autonomy, and resilience, consistent with definitions of stable self-esteem. Dimension B includes items reflecting perceptual, affective, and experiential aspects of body image. Dimension C comprises items assessing belongingness, relational comfort, and interpersonal autonomy. Dimension D contains items related to environmental awareness, perceived environmental threats, and ecological engagement. Dimension E captures perceptions of working conditions, purpose, and the balance between work and personal life. Dimension F includes items reflecting health literacy, access to health resources, and health-related behaviors, consistent with the model's view that knowledge, access, and behavioral engagement jointly underpin physical and psychological wellbeing. Finally, Dimension G includes items that measure positive emotions, engagement, meaning, and future orientation as indicators of existential wellbeing. This structure reflects the theoretical links between each construct and the corresponding item content.

Each item was rated on a 7-point Likert scale, ranging from 1 (“Strongly Disagree” or “Never”) to 7 (“Strongly Agree” or “Always”), depending on the type of question. Higher scores indicate a higher perceived level of wellbeing. An exception is the sleep item in Dimension F, which was assessed using categorical response options reflecting hours of sleep per night and analyzed separately from the Likert-based sub-scales.

In its original version, Dimension B (Body Image) comprised six items reflecting body satisfaction, desire to change one's body, body-shaming experiences in public and private contexts, perceived access to clothing in one's size, and perceived fashion inclusivity. Item content review indicated that the body-shaming items were negatively valenced relative to the intended direction of the subscale and therefore required reverse coding prior to scale scoring. In addition, two items (“Would you change anything in your body?” and “Do you feel that style has no size?”/fashion inclusivity) were judged to be conceptually heterogeneous with the core latent construct of body image and were therefore excluded from the refined version used in the revised analyses. The refined Body Image sub-scale retained four items: body satisfaction/comfort, public body shaming (reverse-coded), private body shaming (reverse-coded), and perceived ability to buy clothes in one's size.

In its original version, Dimension F (Health Knowledge and Behavior) comprised a broad set of items reflecting health literacy, access to care, attitudes, and behaviors. Psychometric analyses revealed very low internal consistency, indicating that the dimension aggregated heterogeneous constructs into a single score. Based on both theoretical considerations and empirical findings, Dimension F was restructured into five sub-domains: preventive health behavior (items 1–3), attitudes toward psychological help (item 4, reverse-coded), body-related physiological perception (item 10), dietary behavior (items 11–15), and sleep behavior (item 16). Reliability analyses were subsequently conducted at the sub-scale level rather than for a single composite score.

To provide a clearer overview of the questionnaire structure, Table 1 summarizes the number of items included in each of the seven wellbeing dimensions, as well as one illustrative example per dimension. This mapping allows readers to understand how each theoretical construct was operationalised in the Holistic Scale of Body and Mental Health and Wellbeing, ensuring transparency in the distribution of items and demonstrating how abstract components of the 7DHW model were translated into concrete, participant-facing questions.

**Table 1:** Overview of the seven wellbeing dimensions, number of items, and example items included in the Holistic Scale of Body and Mental Health and Wellbeing.

Dimensions	Number of items	Examples of items (German/English)	Answer (German/English)
A	13	“Haben Sie Mitgeühl mit sich selbst, wenn Sie Fehler machen?” (“Do you feel compassionate about yourself when you fail?”)	Likert scale 1 (nie/never) - 7 (immer/always)
B	4	“Fühlen Sie sich zufrieden und wohl in Ihrem Körper?” (“Do you feel satisfied and comfortable with your body?”)	Likert scale 1 (nie/never) - 7 (immer/always); Inverted likert scale 1 (immer/always) - 7 (nie/never)
C	8	“Haben Sie das Gefühl, dass Sie bei Ihren engen Freunden Autonomie haben?” (“Do you feel you have autonomy with your close friends?”)	Likert scale 1 (nie/never) - 7 (immer/always)
D	8	“Die Luftqualität in den großen Weltstädten wird sich negativ auf die Gesundheit auswirken.” (“Air quality in major world cosmopolitan cities will negatively impact health.”)	Likert scale 1 (Stimme überhaupt nicht zu/strongly disagree) - 7 (Stimmevoll und ganz zu/strongly agree)
E	11	“Haben Sie das Gefühl, dass Ihre Arbeit einen Sinn für Sie hat?” (“Do you feel your work has a meaningful purpose for you?”)	Likert scale 1 (nie/never) - 7 (immer/always)
F	11	“Haben Sie das Bedürfnis, einmal im Jahr eine Blutanalyse durchzuführen?” (“Do you feel the need to make blood analysis once a year?”)	Likert scale 1 (nie/never) - 7 (immer/always)
G	24	“Haben Sie jemals eine Situation als intrinsisch bereichernd erlebt?” (“Do you ever experience a situation as intrinsically rewarding?”)	Likert scale 1 (nie/never) - 7 (immer/always)

A complete item-level description, including item wording, scoring direction, reverse-coded items, removed items, and the final sub-domain structure, is provided in Table 2.

**Table 2:** Item-level description of the Holistic Scale of Body and Mental Health and Wellbeing (7DHW), including item wording, scoring direction, reverse coding, inclusion status, and final subdomain structure.

Item	Dimension	Sub-domain	Item wording (translated)	Direction	Reverse	Status
B1	Body image	Body satisfaction	Do you feel satisfied with your body?	Positive	No	Included
B2	Body image	Body shaming (public)	Have you experienced body shaming in public?	Negative	Yes	Included
B3	Body image	Body shaming (private)	Have you experienced body shaming in private?	Negative	Yes	Included
B4	Body image	Clothing access	Are you able to find clothes in your size?	Positive	No	Included
B5	Body image	Body change	Would you change anything in your body?	Mixed	No	Removed
B6	Body image	Inclusivity	Do you feel that fashion includes all body sizes?	Mixed	No	Removed
F1	Health knowledge and behavior	Preventive health	Do you attend regular medical check-ups?	Positive	No	Included
F2	Health knowledge and behavior	Preventive health	Do you regularly monitor your health (e.g., blood tests)?	Positive	No	Included
F3	Health knowledge and behavior	Preventive health	Do you take preventive actions to maintain your health?	Positive	No	Included
F4	Health knowledge and behavior	Psychological help (single item)	Would you feel comfortable seeking psychological help if needed?	Positive	No	Included (single-item)
F5	Health knowledge and behavior	Body-related perception (single item)	How do you perceive your metabolism or body functioning?	Neutral	No	Included (single-item)
F6	Health knowledge and behavior	Dietary behavior	Do you avoid sugar in your diet?	Positive	No	Included
F7	Health knowledge and behavior	Dietary behavior	Do you avoid carbohydrates?	Positive	No	Included
F8	Health knowledge and behavior	Dietary behavior	Do you avoid animal-based products?	Positive	No	Included
F9	Health knowledge and behavior	Dietary behavior	Do you consume processed food?	Negative	No	Included
F10	Health knowledge and behavior	Dietary behavior	Do you prefer organic or unprocessed foods?	Positive	No	Included
F11	Health knowledge and behavior	Sleep behavior (single item)	Do you feel that your sleep is sufficient and restorative?	Positive	No	Included (single-item)

Scoring is recommended at the domain/subdomain level rather than as a single total score.

#### 3.2.1 Scoring procedure

Given the multidimensional structure identified in several domains, a global total score for the 7DHW questionnaire is not currently recommended. Instead, scores should be interpreted at the dimension or sub-domain level. For Dimension B, analyses were conducted using the refined four-item version, after reverse coding negatively worded body-shaming items so that higher scores consistently reflected more positive body image and lower perceived stigma. For Dimension F, scores were calculated separately for the preventive health behavior sub-scale (F1) and dietary behavior sub-scale (F4). The single-item indicators attitudes toward psychological help (F2) and body-related perception (F3) were analyzed descriptively and were not included in internal consistency estimation or composite scoring.

### 3.3 Procedure

Participants were recruited in collaboration with ***Company's name (Blind for Review)***, a German company specializing in online rehabilitation and physiotherapy. In agreement with the company, we accessed their database of former clients who had previously consented to be contacted for research and therapeutic development purposes. These individuals were invited via direct email to complete the questionnaire. Participants were offered a voucher for use on the ***Company's name (Blind for Review)*** digital therapy platform as a means of compensating their time. This approach supported participant engagement and resulted in 677 valid responses, surpassing the minimum sample size required for robust psychometric validation. The questionnaire was administered online via a secure data collection platform. All participants provided informed consent prior to participation, and no identifying personal information was collected. The study followed all relevant ethical principles and was conducted in accordance with the General Data Protection Regulation (GDPR) and applicable institutional ethics protocols.

### 3.4 Data analysis

All data were collected via the questionnaire and analyzed according to established psychometric procedures. The following steps were conducted:

1. **Descriptive statistics**. Means, standard deviations, and distributions were computed for all items and demographic variables.
2. **Internal consistency**. Reliability was assessed using Cronbach's α, McDonald's ω and corrected item–total correlations (CITC). Thresholds were α≥0.70 and ω≥0.70 (acceptable) and CITC >0.30. For multidimensional constructs, reliability was assessed at the sub-scale level rather than for a single composite score.
3. **Construct validity (internal structure)**.

- *Exploratory Factor Analysis (EFA):* Exploratory Factor Analyses (EFA) were conducted using the minimum residual (MINRES) extraction method with oblimin rotation, allowing for correlated factors. This approach was selected given the expected conceptual relatedness between sub-domains of holistic wellbeing. Oblimin rotation was preferred over orthogonal rotations to account for the theoretical interdependence between dimensions of wellbeing.
- *Confirmatory Factor Analysis (CFA):* EFAs were followed by CFAs to examine factorial stability and to evaluate the extent to which each dimension could be represented by a unidimensional structure. For each dimension, an initial single-factor model was specified. Where supported by exploratory results, alternative models reflecting correlated factors were considered to better represent the observed structure. CFAs were estimated using robust maximum likelihood (MLR). Model evaluation relied on multiple fit indices: Comparative Fit Index (CFI), Tucker–Lewis Index (TLI), Root Mean Square Error of Approximation (RMSEA) with 90% confidence intervals, and the chi-square test. Cutoffs followed Hu and Bentler's recommendations (CFI/TLI ≥ 0.90 = adequate; RMSEA ≤ 0.08 = acceptable; non-significant χ^2^ desirable but interpreted cautiously due to sample size sensitivity). Standardized factor loadings, standard errors, and *Z*-statistics were inspected to evaluate local fit. Poor fit of the unidimensional model was interpreted as evidence against a single-factor structure, rather than as direct confirmation of a specific multidimensional or hierarchical model.
- *Multidimensional structures:* For Dimension F, which showed clear evidence of multidimensionality, factor analyses were interpreted at the subdomain level rather than imposing a unidimensional model.

**4. Group differentiation (known-groups validity)**. Group comparisons were conducted using independent-samples *t*-tests (Welch correction for unequal variances) for gender and one-way ANOVAs across six age categories (18–30, 31–40, 41–50, 51–60, 61–70, 70+). Significant ANOVAs were followed by Tukey HSD *post hoc* tests. Effect sizes reported were Cohen's *d* (for *t*-tests) and η^2^ (for ANOVAs). For Dimension F, analyses were conducted at the subscale level (e.g., preventive behavior and dietary behavior) rather than using a single composite score.
**5. Assumptions and robustness checks**. Normality was examined via skewness, kurtosis, and the Shapiro–Wilk test; homogeneity of variances was tested with Levene's test. When assumptions were violated, Welch's ANOVA and/or non-parametric alternatives were considered.
**6. Scoring revision and item-level diagnostics**. During revision, item-level diagnostics were conducted for Dimensions B and F, including inspection of item wording, keying direction, corrected item–total correlations, and alpha-if-item-deleted statistics. For Dimension B, two negatively valenced items were reverse-scored, and two conceptually heterogeneous items were removed, yielding a refined four-item sub-scale. For Dimension F, analyses revealed substantial heterogeneity across items, leading to a restructuring into conceptually coherent sub-domains. Reliability and group comparisons were therefore conducted at the sub-scale level rather than for a single composite score.

All statistical tests were two-tailed and conducted using a significance level of *p* < 0.05.

## 4 Results

The results are presented in four steps, corresponding to the main validation objectives. First, exploratory analyses were conducted to examine distributional properties and item behavior. Second, construct validity was assessed through factor analysis and internal consistency estimates. Third, item-level analyses provided additional evidence for reliability and content alignment. Finally, group differentiation analyses were performed using *t*-tests and ANOVAs to evaluate preliminary construct validity across gender and age. Together, these analyses provide a comprehensive assessment of the scale's psychometric robustness.

### 4.1 Exploratory data analysis

To assess the preliminary properties of the questionnaire dataset (*N* = 677), an Exploratory Data Analysis (EDA) was conducted. This analysis included descriptive statistics at the item and dimension levels, identification of missing values, and detection of outliers. The dataset presented a negligible proportion of missing data, with no item showing systematic omission. All dimensions were fully completed by participants, allowing for comprehensive analysis. To provide a structured overview, the questionnaire items were grouped into the seven core dimensions of holistic wellbeing, as defined by the theoretical model:

- Dimension A - Self-Esteem.
- Dimension B - Body Image.
- Dimension C - Friendship and Social Relationships.
- Dimension D - Environment.
- Dimension E - Meaningful Work.
- Dimension F - Health Knowledge and Behavior.
- Dimension G - Sense of Future.

For Dimensions A–E, and G, dimension scores were calculated as the mean of their respective items. Dimension B scores were computed using the refined four-item version. For Dimension F, given its multidimensional structure, descriptive statistics were examined at the sub-scale level rather than using a single composite score. Descriptive statistics indicated the following average scores (mean ± SD):

- Self-Esteem (A): 4.78 ± 0.71.
- Body Image (B, refined): 5.27 ± 1.17.
- Social Relationships (C): 5.44 ± 1.02.
- Environment (D): 5.98 ± 1.01.
- Work (E): 5.01 ± 1.00.
- Health Knowledge and Behavior (F; multidimensional):

- Preventive Health Behavior (F1): 4.21 ± 1.58.
- Attitudes toward Psychological Help (F2): 6.50 ± 1.21.
- Body-related Perception (F3): 4.32 ± 1.63.
- Dietary Behavior (F4): 3.13 ± 0.93.

- Sense of Future (G): 4.59 ± 0.70.

The highest mean score was observed in Environment (D), suggesting strong ecological awareness and engagement with nature-based wellbeing practices. This pattern was followed by Social Relationships (C) and Work (E), indicating robust interpersonal and occupational engagement. Lower mean scores were observed for specific sub-domains within Dimension F, particularly dietary behavior, suggesting potential areas of vulnerability or unmet needs within the sample.

The refined Body Image dimension showed higher mean scores and greater variability than the original version [Body Perception (B): 4.42 ± 0.77], reflecting improved measurement coherence following item recoding and the removal of conceptually heterogeneous items. This suggests that the refined scale provides a more consistent and interpretable assessment of body-related perceptions.

Given the multidimensional structure of Dimension F, descriptive statistics were examined at the sub-scale level rather than using a single composite score. This approach was adopted based on empirical evidence indicating that Dimension F comprises conceptually distinct sub-domains. Additional indicators, including attitudes toward psychological help, body-related perception, and sleep behavior, were analyzed separately.

To assess the presence of extreme cases in participants' responses, an Interquartile Range (IQR) method was applied to each of the seven theoretical dimensions of the questionnaire. This statistical approach defines outliers as those participants whose mean scores fall below *Q1*−1.5 × *IQR* or above *Q3*+1.5 × *IQR*, where Q1 and Q3 represent the first and third quartiles, respectively. In the dimension D - Environment, which includes items related to climate change, ecosystem protection, and nature-based wellbeing rituals, five participants were identified as outliers with mean scores below the IQR threshold. Their average responses ranged from 2.33 to 3.17, substantially lower than the overall mean of the dimension (5.98). No participants exceeded the upper IQR bound, indicating a strong consensus among the majority of the sample regarding high environmental awareness. Across all seven dimensions, outlier analysis using the IQR method revealed a limited number of extreme cases, ranging from 5 (Body Image and selected sub-domains of Dimension F) to 20 (Environment). All data points were retained for subsequent psychometric validation due to their potential relevance and low impact on aggregated results.

While outliers were more common in some dimensions, particularly those involving subjective experience or external context that may vary greatly between individuals, they were not frequent enough to distort central tendency or dispersion measures. Their inclusion also ensures a more ecologically valid representation of wellbeing profiles in diverse populations. For Dimension F, outlier detection was performed separately for each sub-domain.

**Figure 1:**
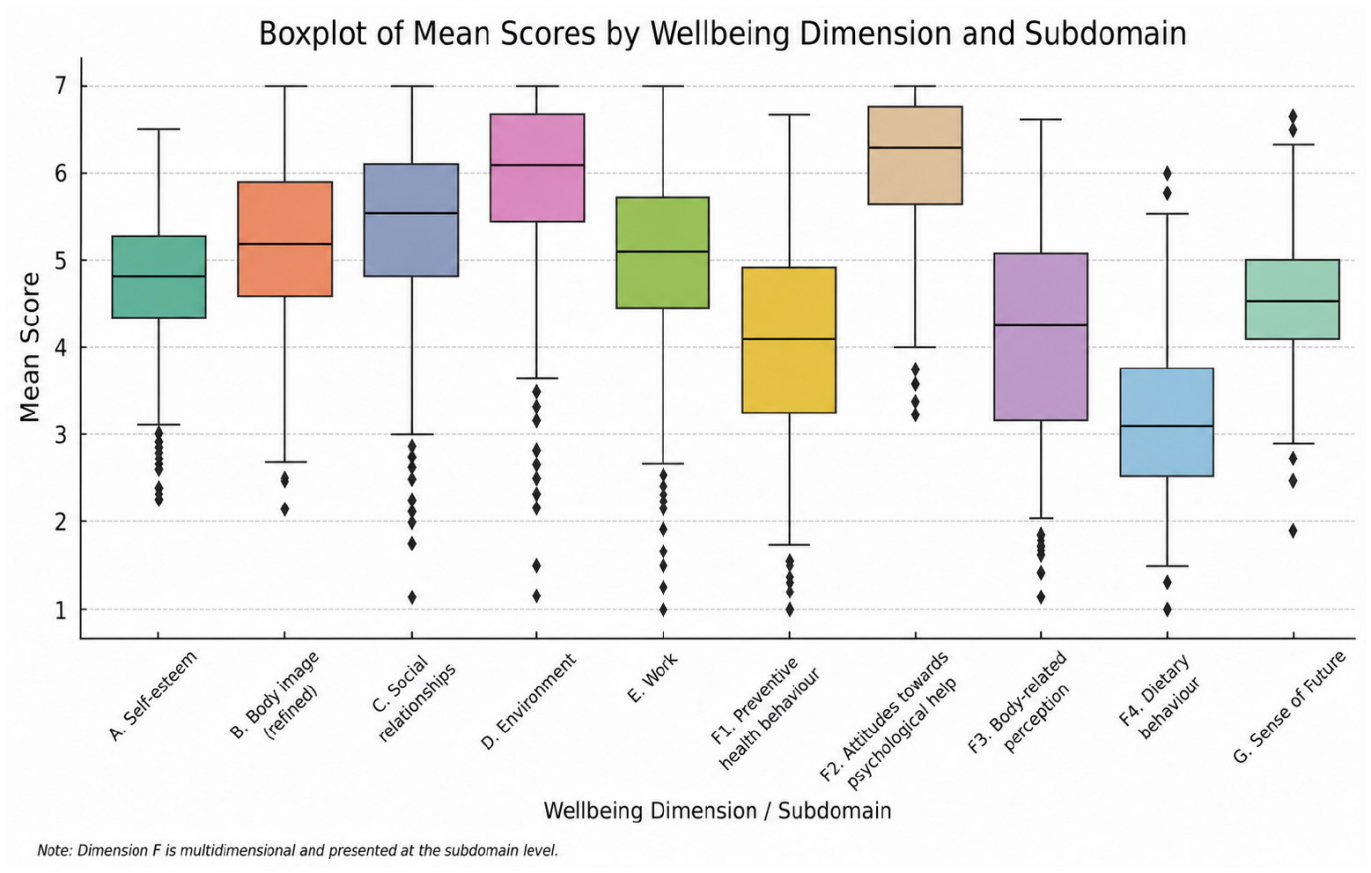
Distribution of mean scores across wellbeing dimensions and sub-domains of the Holistic Scale of Body and Mental Health and Wellbeing. Dimension B reflects the refined four-item Body Image scale. Dimension F is presented at the subdomain level (F1: preventive health behavior; F2: attitudes toward psychological help; F3: body-related perception; F4: dietary behavior) due to its multidimensional structure. The figure is intended solely for descriptive visualization of score distributions and was not used to determine factor retention or factorial structure.

The overall distribution of average scores across the seven dimensions is shown in Figure 1 (Boxplot of Mean Scores). The boxplot confirms the relative consistency in participant responses, with moderate variability observed in dimensions such as C (Social Relationships) and G (Sense of Future). These findings support the adequacy of the data for subsequent reliability and factorial analyses.

### 4.2 Construct validity—Statistical analysis by dimension

Construct validity was examined by evaluating the internal structure of the scale and the coherence of each of its seven theoretically defined dimensions. For each dimension, items were aggregated and subjected to reliability analysis, followed by factor analytic techniques to assess whether the expected latent structure was supported. Cronbach's alpha and McDonald's ω coefficients were computed as indicators of internal consistency, with thresholds of α≥0.70 and ω≥0.70 considered acceptable for research use. Item–total correlations and inter-item statistics were also inspected to verify the homogeneity of each dimension. This multi-step analysis ensured that the scale not only reflected the theoretical model but also demonstrated empirical reliability and validity at the sub-scale level.

#### 4.2.1 Dimension A

An Exploratory Factor Analysis (EFA) using the minimum residual (MINRES) extraction method with oblimin rotation was conducted to examine the underlying structure of Dimension A items from the holistic questionnaire. The Kaiser-Meyer-Olkin (KMO) measure of sampling adequacy was excellent (KMO = 0.908), and Bartlett's test of sphericity was significant (*X*^2^ = 3,990, df = 66, *p* < 0.001), indicating suitability for factor analysis. Four factors were extracted based on eigenvalues greater than one and theoretical interpretability of the factor solution, accounting for 59.2% of the total variance. These factors captured dimensions of (1) self-perception and self-confidence, (2) self-awareness and relational skills, (3) autonomy and boundary-setting, and (4) self-acceptance and resilience in the face of adversity. All items loaded significantly onto at least one factor, with most factor loadings exceeding 0.60 and low uniqueness, demonstrating strong construct representation.

Model fit indices further supported the adequacy of the four-factor solution. The root mean square error of approximation (RMSEA) was 0.0314 (90% CI: 0.0122–0.0481), below the conventional threshold of 0.06, and the Tucker-Lewis Index (TLI) was 0.989, indicating excellent model fit. Although the chi-square test reached significance (*X*^2^ = 40.0, df = 24, *p* = 0.021), this is likely influenced by sample size sensitivity and does not detract from the overall fit. Inter-factor correlations ranged from 0.556 to 0.674, suggesting moderately strong relationships among constructs while maintaining discriminant validity. These findings provide initial evidence of a coherent factorial structure at the exploratory level, suggesting that Dimension A comprises several related but distinct components of psychological functioning.

Building on the empirical four-factor solution identified in the exploratory phase, a Confirmatory Factor Analysis (CFA) was conducted to test the factorial stability and theoretical coherence of Dimension A. This step allowed us to evaluate whether the proposed structure, comprising self-perception/confidence, self-awareness/relational skills, autonomy/boundary-setting, and self-acceptance/resilience, adequately reproduced the covariance patterns observed in the data.

The four-factor CFA model showed good local fit, with all items loading significantly on their respective latent constructs (standardized loadings ranging from 0.48 to 0.92, *p* < 0.001), indicating strong convergent validity. However, the global model fit indices for the single higher-order factor solution indicated limitations (CFI = 0.835, TLI = 0.799, RMSEA = 0.134), suggesting that a strictly unidimensional structure cannot fully capture the complexity of the Dimension A construct. The chi-square statistic was also significant (*X*^2^ = 218, *df* = 20, *p* < 0.001). A confirmatory factor analysis (CFA) was conducted to evaluate whether Dimension A could be adequately represented by a unidimensional (single-factor) model. Although all items loaded significantly on the latent factor, the global fit indices indicated poor model fit (CFI = 0.835, TLI = 0.799, RMSEA = 0.134), suggesting that a unidimensional representation is not supported.

Importantly, this result should be interpreted as evidence against unidimensionality, rather than as direct confirmation of a specific multidimensional or hierarchical structure. In combination with the EFA findings, the results suggest that Dimension A is better conceptualized as a set of related but distinct subcomponents, reflecting multiple facets of psychological functioning. However, alternative CFA models (e.g., correlated-factor or higher-order structures) were not formally tested and should be examined in future research.

Taken together, the EFA and CFA results suggest that Dimension A is unlikely to be adequately represented as a unidimensional construct. While the EFA indicates a clear multidimensional structure, the CFA results provide supporting evidence by demonstrating that a single-factor model does not fit the data well. These findings suggest that Dimension A may involve multiple related components and is not adequately represented by a unidimensional model.

The statistical analysis of Dimension A suggests a four-factor structure aligned with the theoretical constructs of self-perception, self-awareness, autonomy, and coping with adversity. These dimensions are empirically distinct yet moderately interrelated, validating their role in assessing holistic psychological functioning. Overall, the results indicate that Dimension A shows promising psychometric properties, supporting its continued development and further validation in independent samples.

#### 4.2.2 Dimension B

An exploratory factor analysis (EFA) was performed on Dimension B items using the minimum residual extraction method with oblimin rotation to evaluate the underlying factor structure related to perceptions of body image and inclusivity. The KMO measure of sampling adequacy was acceptable (KMO = 0.694), and Bartlett's test of sphericity was significant (*X*^2^ = 865, *df* = 10, *p* < 0.001), confirming the suitability of the data for factor analysis. Two factors emerged with eigenvalues above one and theoretical interpretability of the factor structure, explaining 50.3% of the total variance (Factor 1: 29.6%; Factor 2: 20.7%). Factor 1 captured experiences related to body shaming (for example, private and public experiences, as well as perceived ability to buy suitable clothing), while Factor 2 reflected positive body satisfaction and perceptions of inclusivity. All items showed moderate to high loadings (for example, 0.949 for private body shaming and 0.723 for public body shaming), with acceptable levels of uniqueness.

In the initial six-item version, however, Dimension B showed severe internal consistency problems. Inspection of the CFA results further suggested a strong polarity in item loadings, with body-shaming items loading in the opposite direction to positively worded body image items. This pattern indicated a likely scoring/keying issue rather than support for a coherent unidimensional or even clearly interpretable multidimensional scale.

Although the exploratory solution suggested a two-factor structure with acceptable statistical fit (RMSEA = 0.00, 90% CI: 0.00–0.0752; TLI = 1.01; *X*^2^ = 0.156, *df* = 1, *p* = 0.693), these findings must be interpreted with caution. The combination of very low reliability estimates and opposing item polarity suggests that the observed structure may reflect methodological artifacts rather than a stable latent construct.

A subsequent confirmatory factor analysis (CFA) was conducted to further evaluate the structure of Dimension B and to test whether a unidimensional model could adequately represent body-related perceptions. Although all items showed significant loadings on a single latent factor (standardized estimates ranging from –1.39 to 0.92, all *p* < 0.001), the overall model fit was poor (CFI = 0.888, TLI = 0.776, RMSEA = 0.169, 90% CI: 0.141–0.198; *X*^2^ = 101, *df* = 5, *p* < 0.001). The pattern of loadings again revealed a clear divergence, with body-shaming items loading negatively and positively framed body image items loading positively. This pattern further indicates that the original item set combined indicators with opposing directional meaning that had not been aligned during scoring.

We therefore re-examined item polarity and recoded the two body-shaming items so that higher scores consistently reflected more positive body image (i.e., fewer body-shaming experiences). In addition, two items—body change (“Would you change anything in your body?”) and fashion inclusivity (“Do you feel that style has no size?”) were identified as conceptually heterogeneous and only weakly aligned with the intended latent construct. These items were therefore removed. The refined four-item version of Dimension B, comprising body satisfaction/comfort, public body shaming (reverse-coded), private body shaming (reverse-coded), and perceived ability to buy clothes in one's size, was then re-analyzed.

To evaluate whether the revised item set provided a more coherent measurement model, the refined four-item version of Dimension B was re-analyzed. Internal consistency improved substantially compared to the original version (Cronbach's α = 0.756, McDonald's ω = 0.758). Corrected item-total correlations were all within acceptable ranges (range = 0.462–0.644), indicating improved homogeneity across items. While these results indicate acceptable internal consistency, reliability alone does not guarantee factorial validity, and the dimensional structure should therefore be interpreted with caution.

A confirmatory factor analysis (CFA) of the refined four-item model showed improved alignment of item loadings relative to the original six-item solution, with all items loading positively and significantly on the latent factor (standardized loadings ranging from 0.450 to 0.848, all *p* < 0.001), indicating that reverse coding successfully aligned item polarity. However, global fit indices remained mixed (CFI = 0.937, TLI = 0.811, RMSEA = 0.191, 90% CI: 0.000–0.238, *X*^2^ = 51.48, *df* = 2, *p* < 0.001), suggesting that a strictly unidimensional representation is not fully supported. These results indicate that, although the refined scale is substantially more coherent than the original version, its factorial structure should be considered provisional and requires confirmation in independent samples.

These results suggest that the refined four-item version provides a more internally coherent and theoretically focused representation of body image compared to the original item set, while still warranting cautious interpretation in future validation work.

Taken together, the initial analyses do not support substantive interpretation of the original six-item version of Dimension B as a psychometrically adequate sub-scale. Instead, they highlight the importance of item polarity alignment and conceptual coherence in scale development. The refined four-item version represents a substantially improved and internally coherent measure of body-related comfort and reduced body-related stigma. However, given the mixed CFA fit indices, its factorial structure should be considered provisional, and further validation in independent samples is required.

Details of item refinement are provided in Table 2.

#### 4.2.3 Dimension C

An EFA was conducted for Dimension C items using the minimum residual method with oblimin rotation to assess the latent structure underlying interpersonal connectedness and autonomy in social relationships. Sampling adequacy was high, as indicated by a global KMO value of 0.878, with item-level KMOs ranging from 0.846 to 0.944. Bartlett's test of sphericity confirmed factorability of the correlation matrix (*X*^2^ = 3797, *df* = 28, *p* < 0.001). Based on eigenvalues above one, three factors were extracted, explaining 74.3% of the total variance (Factor 1: 27.8%, Factor 2: 25.4%, Factor 3: 21.1%). Items showed strong loadings onto their respective factors, with particularly high values for “I feel I have close friends” (0.959), “Do you feel comfortable giving in a relation?” (0.951), and “Do you feel comfortable sharing in a relation?” (0.888).

The factor structure revealed conceptually distinct yet interrelated dimensions: (1) emotional closeness and supportiveness in relationships, (2) sense of belonging and connection to a community, and (3) perceived autonomy in social settings. Factor correlations ranged from 0.625 to 0.648, supporting a moderately correlated but distinguishable multidimensional model. Model fit indices indicated an adequate fit to the data (RMSEA = 0.0826, 90% CI: 0.0586–0.109; TLI = 0.966; BIC = –6.34), though the RMSEA was slightly above the conventional cutoff, potentially reflecting the complexity of social-relational constructs and the limited degrees of freedom (*X*^2^ = 39.3, *df* = 7, *p* < 0.001). Overall, the EFA suggested an interpretable factor structure for Dimension C, with all items demonstrating adequate communalities and meaningful loading patterns.

A subsequent CFA was conducted to evaluate whether Dimension C could be adequately represented by a unidimensional (single-factor) model, and to examine the extent to which the multidimensional structure suggested by the EFA could be simplified. Although all items displayed strong and statistically significant factor loadings (standardized estimates ranging from 0.91 to 1.30, all *p* < 0.001), the unidimensional model demonstrated poor global fit (CFI = 0.845, TLI = 0.784, RMSEA = 0.208, 90% CI: 0.194–0.223). The highly significant chi-square statistic (*X*^2^ = 607, *df* = 20, *p* < 0.001) and poor global fit indices (CFI = 0.845, TLI = 0.784, RMSEA = 0.208) indicate that a unidimensional model is not supported for Dimension C. Importantly, this result should be interpreted as evidence against a single-factor structure, rather than as direct confirmation of a specific multidimensional model. In combination with the EFA findings, the results suggest that emotional closeness, community belonging, and relational autonomy represent related but distinct components of interpersonal functioning. However, alternative CFA models (e.g., correlated-factor or higher-order models) were not formally tested in the present study and should be examined in future research. These findings highlight the conceptual distinction between relational closeness, social connectedness, and interpersonal autonomy, suggesting that Dimension C is better understood as a multidimensional construct.

Taken together, the EFA and CFA results indicate that Dimension C is unlikely to be adequately represented as a unidimensional construct. While the EFA suggests a clear multidimensional structure, the CFA findings provide supporting evidence by demonstrating poor fit of a single-factor model. These results suggest that Dimension C is unlikely to be adequately represented as unidimensional. Although further confirmatory analyses are needed to explicitly test alternative structural models. Overall, Dimension C demonstrates strong internal consistency and promising construct validity, supporting its use in the assessment of social and relational wellbeing, while warranting further structural validation.

#### 4.2.4 Dimension D

An EFA using the minimum residual extraction method and oblimin rotation was applied to Dimension D to explore the factorial structure underlying attitudes and beliefs related to climate change, environmental health, and nature-based practices. Sampling adequacy was high, with a global KMO of 0.881 and item-level KMOs ranging from 0.833 to 0.920, supporting the suitability of the data for factor analysis. Bartlett's test of sphericity was highly significant (*X*^2^ = 2877, *df* = 28, *p* < 0.001), confirming sufficient inter-item correlations. Four factors were extracted, based on eigenvalues greater than one and the boxplot in Figure 1, accounting for 67.8% of the total variance (Factor 1: 26.9%, Factor 2: 16.0%, Factor 3: 13.4%, Factor 4: 11.5%). Items such as “Sea-level rise will impact habitability” (loading = 1.000) and “Climate changes are affecting the circulation of microorganisms...” (loading = 1.000) exhibited strong saturation, while others showed moderate contributions and adequate communalities.

The factor structure suggests four distinct yet interrelated components: (1) perceived impacts of sea-level rise, (2) environmental health threats (for example, air quality, and heat-related stress), (3) ecosystem-based mitigation beliefs, and (4) personal engagement in nature-based activities. Inter-factor correlations ranged from r = 0.390 to r = 0.683, indicating moderate to strong associations between constructs. Model fit was acceptable though slightly marginal, with RMSEA = 0.114 (90% CI: 0.0717–0.163), TLI = 0.913, and BIC = 6.58. The chi-square statistic was significant (*X*^2^ = 19.6, *df* = 2, *p* < 0.001), likely due to the small degrees of freedom. Despite this, the factorial solution appears theoretically interpretable and warrants further confirmatory investigation, particularly given the environmental and sociopsychological diversity of the items assessed.

A subsequent CFA was conducted to evaluate whether Dimension D could be adequately represented by a unidimensional (single-factor) model, and to examine whether the multidimensional structure suggested by the EFA could be simplified. Although all items loaded significantly on the latent factor (standardized loadings ranging from 0.36 to 1.36, all *p* < 0.001), the unidimensional model demonstrated suboptimal global fit (CFI = 0.931, TLI = 0.903, RMSEA = 0.121, 90% CI: 0.107–0.136). The RMSEA value in particular exceeded conventional thresholds, indicating notable misfit despite generally strong local item performance. The significant chi-square statistic (*X*^2^ = 218, *df* = 20, *p* < 0.001) further underscores the mismatch between the observed covariance structure and a single-factor solution. Inspection of the factor loadings suggests that items related to climate change impacts, environmental health threats, and ecosystem-based mitigation beliefs cluster strongly, whereas items reflecting nature-based practices or personal engagement contribute less to the overarching construct. Inspection of the factor loadings indicates that items related to climate change impacts, environmental health threats, and ecosystem-based mitigation beliefs contribute more strongly to the latent factor, whereas items reflecting nature-based practices or personal engagement contribute less.

Importantly, these results should be interpreted as evidence against a unidimensional representation, rather than as direct confirmation of a specific multidimensional structure. In combination with the EFA findings, the results suggest that Dimension D reflects multiple related but distinct components of environmental and ecopsychological functioning. However, alternative CFA models (e.g., correlated-factor or higher-order structures) were not formally tested and should be examined in future research. Taken together, the EFA and CFA results indicate that Dimension D is unlikely to be adequately represented as a unidimensional construct. While the EFA suggests a multidimensional structure capturing climate-impact perceptions, environmental health concerns, ecological beliefs, and nature-oriented engagement, the CFA findings provide supporting evidence by demonstrating poor fit of a single-factor model. These results suggest the presence of multiple related components of environmental wellbeing, although further confirmatory analyses are needed to explicitly test alternative structural models. Overall, Dimension D demonstrates strong internal consistency and promising construct validity, supporting its use as a measure of environmental and ecopsychological functioning, while warranting further structural validation. The CFA findings further indicate that a unidimensional representation is not sufficient to capture the complexity of this dimension.

#### 4.2.5 Dimension E

An EFA using the minimum residual extraction method and oblimin rotation was conducted to investigate the factorial structure of Dimension E, which focuses on perceptions of workplace wellbeing and environmental conditions. The overall sampling adequacy was good, as reflected in a global KMO value of 0.832, with item-level KMOs ranging from 0.548 to 0.925. Bartlett's test of sphericity was statistically significant (*X*^2^ = 2857, *df* = 45, *p* < 0.001), supporting the factorability of the correlation matrix. The boxplot in Figure 1 and eigenvalue criteria suggested a four-factor solution, explaining 59.2% of the total variance (Factor 1: 18.5%, Factor 2: 16.2%, Factor 3: 15.3%, Factor 4: 9.3%). Items showed substantial factor loadings, particularly for organizational/spatial conditions (0.898), healthy working conditions (0.896), and perceived meaning of work for others (0.905).

The factors delineated in this analysis reflect four conceptual domains: (1) physical and organizational workplace infrastructure, (2) perceived meaningfulness and inclusivity of work, (3) work-life balance and financial wellbeing, and (4) physical presence and office engagement. Inter-factor correlations ranged from negligible (r = 0.00019) to moderate (r = 0.743), indicating a partially independent but integrative multidimensional structure. Model fit indices demonstrated satisfactory adequacy, with RMSEA = 0.0411 (90% CI: 0.0175–0.0642), TLI = 0.982, and a BIC of –48.1. Although the chi-square test was significant (*X*^2^ = 23.6, *df* = 11, *p* < 0.015), this is likely attributable to sample size effects rather than poor model fit. These results provide preliminary support for the structural coherence of the item set and validate the theoretical model underlying this domain.

A subsequent CFA was conducted to evaluate whether Dimension E could be adequately represented by a unidimensional (single-factor) model, and to assess whether the multidimensional structure suggested by the EFA could be simplified. Although all items loaded significantly onto the single latent factor (standardized loadings ranging from 0.07 to 1.40, with most *p* < 0.001), the global fit indices indicated substantial model misfit (CFI = 0.739, TLI = 0.665, RMSEA = 0.177, 90% CI: 0.166–0.188). The chi-square statistic was also significant and notably large (*X*^2^ = 774, *df* = 35, *p* < 0.001), reinforcing the inadequacy of the unidimensional solution. Inspection of the factor loadings revealed that items related to organizational infrastructure, financial wellbeing, meaningfulness of work, and hybrid working arrangements contributed differently to the general factor, with some items showing weaker associations (e.g., physical presence and office engagement). Importantly, these findings should be interpreted as evidence against a unidimensional representation, rather than as direct confirmation of a specific multidimensional structure. In combination with the EFA results, the findings suggest that Dimension E reflects multiple related but distinct components of workplace wellbeing. However, alternative CFA models (e.g., correlated-factor or higher-order structures) were not formally tested and should be examined in future research.

Taken together, the EFA and CFA results indicate that Dimension E is unlikely to be adequately represented as a unidimensional construct. The EFA suggests a multidimensional structure encompassing environmental, relational, financial, and experiential aspects of workplace wellbeing, while the CFA provides supporting evidence by demonstrating poor fit of a single-factor model. These results suggest the presence of multiple related components of occupational wellbeing, although further confirmatory analyses are needed to explicitly test alternative structural models. Overall, Dimension E demonstrates acceptable internal consistency and good construct coherence, supporting its use as a measure of workplace-related wellbeing, while warranting further structural validation. These results provide initial support for the construct coherence and theoretical relevance of Dimension E within the present sample.

#### 4.2.6 Dimension F

An exploratory factor analysis (EFA) was conducted on Dimension F to assess the latent structure of items related to health behaviors, dietary patterns, and psychological attitudes toward care. The analysis employed the minimum residual extraction method with oblimin rotation. Sampling adequacy was moderate (KMO = 0.622), and Bartlett's test of sphericity was significant (*X*^2^ = 1, 660, *df* = 91, *p* < 0.001), supporting the suitability of the data for factor analysis.

The EFA results indicated a complex, heterogeneous structure, with multiple factors emerging, accounting for 41.3% of the total variance. Rather than supporting a single coherent latent construct, the pattern of factor loadings suggested that the items captured several conceptually distinct domains, including preventive health behavior, dietary practices, attitudes toward psychological care, food-related practices, and body-related self-perception. Inter-factor correlations were generally weak to moderate, indicating that these domains are only partially related.

A confirmatory factor analysis (CFA) was subsequently conducted to evaluate whether a unidimensional model could adequately represent Dimension F. The model showed poor fit (CFI = 0.376, TLI = 0.263, RMSEA = 0.138), indicating that a single latent factor does not adequately reproduce the observed covariance structure. Inspection of factor loadings further revealed substantial heterogeneity, with some items loading weakly or in opposing directions, reflecting differences in conceptual meaning across items.

Because Dimension F was restructured into conceptually distinct sub-domains, a correlated two-factor CFA was estimated for the two multi-item sub-scales: preventive health behavior (F1) and dietary behavior (F4). Single-item indicators, including attitudes toward psychological help (F2), body-related perception (F3), and sleep behavior (F5), were not included in the latent model.

A correlated two-factor CFA model was estimated for the two multi-item sub-domains of Dimension F: preventive health behavior (F1; items 1–3) and dietary behavior (F4; items 11–15). However, it should be noted that the preventive health behavior sub-scale demonstrated only modest internal consistency, and the dietary behavior sub-scale remained below conventional reliability thresholds, indicating that these sub-domains should be interpreted with caution. The model showed acceptable fit, χ^2^(19) = 59.57, *p* < 0.001, CFI = 0.958, TLI = 0.938, RMSEA = 0.056, 90% CI [0.040, 0.073]. The correlation between the two latent factors was weak (*r* = 0.20), indicating that preventive health behavior and dietary behavior are related but empirically distinct sub-domains.

Taken together, these findings indicate that Dimension F cannot be adequately represented as a unidimensional scale.

Standardized loadings ranged from 0.23 to 0.88. Loadings were generally stronger for preventive health behavior (0.23–0.88) than for dietary behavior (0.22–0.80), indicating that the dietary sub-domain remains more heterogeneous. While these results support interpreting Dimension F as an exploratory multidimensional domain rather than as a single composite scale. In particular, the relatively low internal consistency of the dietary behavior sub-scale suggests that further refinement of item content is needed.

The item-to-sub-domain structure is detailed in Table 2.

#### 4.2.7 Dimension G

An EFA was conducted for Dimension G to assess the structure of items measuring components of psychological flourishing, including positive emotions, engagement, flow, relationships, meaning, and accomplishment. The analysis utilized the minimum residual extraction method with oblimin rotation. Sampling adequacy was excellent, as indicated by a global KMO value of 0.906, with item-level KMOs ranging from 0.745 to 0.945. Bartlett's test of sphericity was highly significant (*X*^2^ = 5313, *df* = 231, *p* < 0.001), confirming sufficient inter-item correlations. Six factors were extracted based on eigenvalues above one, explaining a cumulative variance of 47.03% (Factor 1: 9.31%, Factor 2: 9.14%, Factor 3: 8.43%, Factor 4: 8.22%, Factor 5: 6.99%, Factor 6: 4.94%).

The factors appeared conceptually consistent with core dimensions of Seligman's PERMA model. Factor 1 reflects “Positive Emotions,” Factor 2 relates to “Engagement” and skill development, Factor 3 covers “Positive Relationships,” Factor 4 addresses “Meaning” and mentoring, Factor 5 captures “Accomplishment,” and Factor 6 aggregates distinct aspects of “Flow,” including focused concentration, loss of self-awareness, distorted time perception, and the balance between challenge and skill. Model fit indices demonstrated robust adequacy: RMSEA = 0.0428 (90% CI: 0.0359–0.0499), TLI = 0.943, and BIC = –487. The chi-square statistic was significant (*X*^2^ = 256, *df* = 114, *p* < 0.001), likely due to the sample size; however, other indices support the model's validity. Inter-factor correlations ranged from 0.067 to 0.503, indicating moderate but conceptually coherent associations among dimensions of wellbeing.

A subsequent CFA was conducted to evaluate whether Dimension G could be adequately represented by a unidimensional (single-factor) model, and to examine whether the multidimensional structure suggested by the EFA could be simplified. Although most items demonstrated statistically significant loadings in the single-factor model (standardized loadings ranging from –0.41 to 1.21, with the majority *p* < 0.001), global fit indices revealed substantial misfit (CFI = 0.707, TLI = 0.679, RMSEA = 0.096, 90% CI: 0.0919–0.100). The chi-square statistic was large and significant (*X*^2^ = 1, 826, *df* = 252, *p* < 0.001), further indicating that a unidimensional structure is insufficient to reproduce the observed covariance among items covering positive emotions, engagement, relationships, meaning, accomplishment, and flow. Inspection of the factor loadings showed considerable heterogeneity, including several negatively loading items (e.g., self-consciousness and loss of control in flow experiences), which conceptually diverge from more positively valenced wellbeing indicators. Importantly, these findings should be interpreted as evidence against a unidimensional representation, rather than as direct confirmation of a specific multidimensional structure. In combination with the EFA results, the findings suggest that psychological flourishing, as measured in Dimension G, comprises multiple related but distinct components, including positive emotions, engagement, relationships, meaning, accomplishment, and flow. However, alternative CFA models (e.g., correlated-factor or higher-order structures) were not formally tested and should be examined in future research.

Taken together, the EFA and CFA results indicate that Dimension G is unlikely to be adequately represented as a unidimensional construct. While the EFA suggests a multidimensional structure encompassing key components of psychological flourishing, the CFA findings provide supporting evidence by demonstrating poor fit of a single-factor model. These findings suggest that Dimension G captures multiple related aspects of psychological flourishing and is not adequately represented by a unidimensional model. Although further confirmatory analyses are required to explicitly test alternative structural models. Overall, Dimension G demonstrates strong internal consistency and promising construct validity, supporting its use in the assessment of psychological flourishing, while warranting further structural validation. These findings provide initial support for the conceptual coherence and relevance of Dimension G within the present sample.

### 4.3 Internal consistency and item analysis

**Figure 2:**
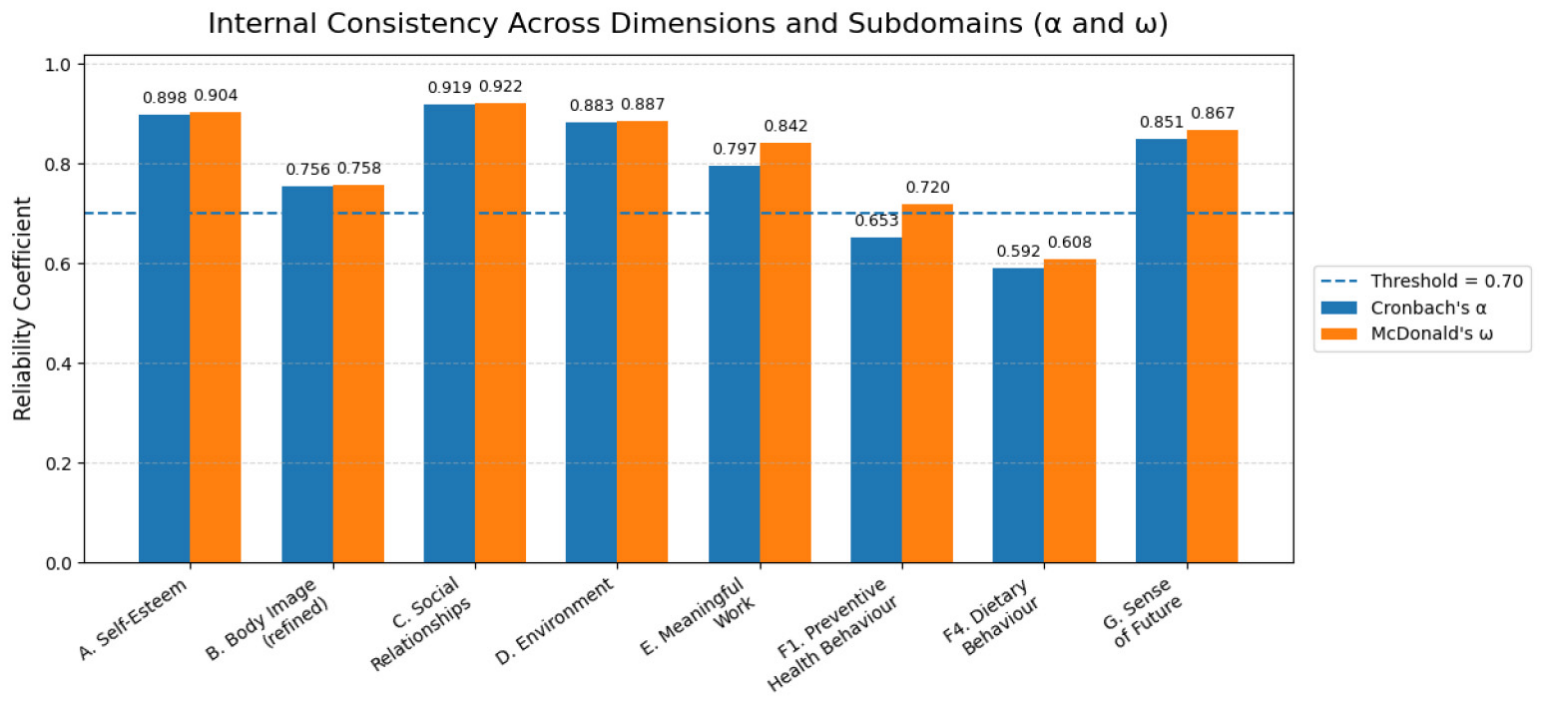
Internal consistency across dimensions and sub-domains of the Holistic Scale of Body and Mental Health and Wellbeing, assessed using Cronbach's α and McDonald's ω. Dimensions A, C, D, and G demonstrated strong reliability (α, ω≥0.85), and Dimension E showed acceptable reliability (α = 0.797, ω = 0.842). Dimension B refers to the refined four-item Body Image scale, which showed improved internal consistency following item recoding and refinement (α = 0.756, ω = 0.758). Dimension F is presented at the sub-domain level, reflecting its multidimensional structure. The preventive health behavior sub-scale (F1) showed acceptable reliability (α = 0.653, ω = 0.720), while the dietary behavior sub-scale (F4) demonstrated moderate reliability (α = 0.592, ω = 0.608). Attitudes toward psychological help (F2) and body-related perception (F3) were measured using single items and are therefore not included in internal consistency estimation. The dashed line indicates the recommended reliability threshold of 0.70.

The internal consistency of the questionnaire was evaluated using Cronbach's α, McDonald's ω, and corrected item–total correlations (CITC). As shown in Figure 2, most sub-scales achieved good to excellent reliability, with α and ω values above the recommended threshold of 0.70. Specifically, strong internal consistency was observed for **Dimension A – Stable Self-Esteem** (α = 0.898, ω = 0.904), **Dimension C – Social Relationships** (α = 0.919, ω = 0.922), **Dimension D – Environment** (α = 0.883, ω = 0.887), and **Dimension G – Sense of Future** (α = 0.851, ω = 0.867). The sub-scale **Dimension E – Meaningful Work** (α = 0.797, ω = 0.842) also demonstrated acceptable internal consistency.

In the initial analysis, **Dimension B – Body Image** showed extremely low reliability (α = 0.012, ω = 0.360), indicating substantial measurement issues. Following item-level diagnostics, including inspection of item polarity and conceptual coherence, two items were reverse-coded, and two conceptually heterogeneous items were removed. The refined four-item version demonstrated substantially improved internal consistency (α = 0.756, ω = 0.758), supporting its use as a coherent measure of body-related comfort and reduced stigma.

Similarly, **Dimension F – Health Knowledge and Behavior** showed poor reliability in its initial form (α = 0.379, ω = 0.411), reflecting the aggregation of heterogeneous constructs into a single score. Based on these findings, Dimension F was restructured into conceptually distinct sub-domains. Reliability was therefore assessed at the sub-scale level rather than for a single composite score. The preventive health behavior sub-scale (F1) demonstrated acceptable internal consistency (α = 0.653, ω = 0.720), while the dietary behavior sub-scale (F4) showed moderate reliability (α = 0.592, ω = 0.608), indicating some remaining heterogeneity. Single-item indicators, including attitudes toward psychological help (F2), body-related perception (F3), and sleep behavior (F5), were retained for descriptive purposes but were not included in latent modeling or internal consistency estimation, and should not be interpreted as psychometrically validated sub-scales.

Taken together, these results indicate that, while most instrument dimensions demonstrate strong internal consistency, Dimensions B and F require refinement. The revised Body Image scale and the sub-domain-based approach to Dimension F provide a more psychometrically coherent representation of these constructs.

The CITC analysis provided more detailed insights into the contribution of individual items. In the reliable sub-scales (Dimensions A, C, D, E, and G), the majority of items exceeded the conventional 0.30 threshold, indicating satisfactory alignment with their respective constructs. For example, in the Dimension C – Social Relationships sub-scale, all items displayed strong corrected item–total correlations (>0.69), supporting the coherence of this dimension. Similarly, items in the Dimension A – Stable Self-Esteem and Dimension G – Sense of Future sub-scales showed consistently high correlations, reinforcing their internal validity. The Dimension E – Meaningful Work sub-scale revealed more heterogeneous item performance, with several items showing strong CITC values (>0.60), while weaker items (< 0.30) may benefit from further refinement.

In the initial version of **Dimension B – Body Image**, almost all items displayed low corrected item–total correlations, confirming a lack of internal coherence. Item inspection and factor-analytic results indicated that this was primarily due to a polarity issue: negatively worded body-shaming items were not aligned with the scoring direction of positively framed body image items. Following this diagnosis, the two body-shaming items were reverse-coded, and two conceptually heterogeneous items (body change and fashion inclusivity) were removed. The refined four-item version of Dimension B showed substantially improved internal consistency (Cronbach's α = 0.756, McDonald's ω = 0.758), with corrected item–total correlations ranging from 0.462 to 0.644, indicating improved homogeneity and construct coherence.

In contrast, the low internal consistency initially observed in **Dimension F – Health Knowledge and Behavior** (α = 0.379, ω = 0.411) reflects a broader conceptual issue: the aggregation of heterogeneous constructs (e.g., preventive health behavior, dietary practices, attitudes toward psychological help, and body-related perceptions) into a single scale. Following this diagnosis, Dimension F was restructured into conceptually coherent sub-domains. At the sub-scale level, reliability estimates improved and became more interpretable. The preventive health behavior sub-scale (F1; items 1–3) demonstrated acceptable internal consistency for a short scale (Cronbach's α = 0.653, McDonald's ω = 0.720), while the dietary behavior subscale (F4; items 11–15) showed lower but still interpretable reliability (Cronbach's α = 0.592, McDonald's ω = 0.608), consistent with the conceptual breadth of dietary practices. Single-item indicators, including attitudes toward psychological help (F2) and body-related perception (F3), were retained for descriptive purposes but were not included in internal consistency estimation.

Taken together, these results demonstrate that most domains of the Holistic Wellbeing Questionnaire show strong or acceptable internal consistency, particularly Dimensions A, C, D, and G, with Dimension E showing acceptable reliability. While Dimension B initially exhibited critically low reliability, subsequent item-level diagnostics and refinement substantially improved its psychometric properties. For Dimension F, the results highlight its multidimensional nature and support a sub-domain-based approach to measurement, rather than a single composite scale. Overall, these findings support the interpretation of the present study as a validation-and-refinement effort of all dimensions, highlighting both the instrument's strengths.

### 4.4 Summary of psychometric findings

To provide an integrative overview of the psychometric performance of each dimension, Table 3 summarizes the factor structure, internal consistency, and overall assessment across all domains of the instrument.

**Table 3:** Summary of psychometric findings across dimensions of the Holistic Scale of Body and Mental Health and Wellbeing, including factor structure, internal consistency, and overall evaluation.

Dimension	Structure (EFA/CFA)	Reliability	Status	Interpretation
A (Self-Esteem)	EFA: 4 factors; CFA: unidimensional model not supported	Strong (α = 0.898, ω = 0.904)	Supported	Coherent construct with strong internal consistency; evidence suggests multiple related subcomponents, though multidimensional structure was not confirmed.
B (Body Image, refined)	EFA: 2 factors (original); CFA: unidimensional model not fully supported (refined)	Acceptable (α = 0.756, ω = 0.758)	Improved (provisional)	Substantially improved after reverse coding and item removal; internally coherent, but factorial structure remains provisional.
C (Social Relationships)	EFA: 3 factors; CFA: unidimensional model not supported	Strong (α = 0.919, ω = 0.922)	Supported	Highly consistent domain capturing multiple aspects of relational functioning; results suggest the construct may not be adequately represented as unidimensional, though alternative CFA models were not tested.
D (Environment)	EFA: 4 factors; CFA: unidimensional model not supported	Strong (α = 0.883, ω = 0.887)	Supported	Promising environmental construct with multiple related components; unidimensional representation insufficient, suggesting multidimensional structure.
E (Meaningful Work)	EFA: 4 factors; CFA: unidimensional model not supported	Acceptable (α = 0.797, ω = 0.842)	Supported (with heterogeneity)	Conceptually coherent but heterogeneous domain; evidence suggests multiple underlying components of workplace wellbeing.
F (Health Knowledge & Behavior)	EFA: heterogeneous; CFA: unidimensional model not supported; 2-factor CFA (F1–F4) acceptable	Mixed (F1: α = 0.653, F4: α = 0.592)	Needs refinement (exploratory)	Not a unidimensional construct; better interpreted as a set of exploratory subdomains. Subscales show limited reliability and should not be treated as stable standalone measures.
G (Sense of Future)	EFA: 6 factors; CFA: unidimensional model not supported	Strong (α = 0.851, ω = 0.867)	Supported	Complex but internally consistent domain reflecting multiple components of psychological flourishing; multidimensional structure suggested but not confirmed.

These results highlight that, while several dimensions are psychometrically robust, others require refinement or reconceptualization, supporting the interpretation that several dimensions may involve multiple related subcomponents rather than strictly unidimensional structures.

### 4.5 Differentiation analysis

To examine known-groups validity and the ability of the Holistic Scale of Body and Mental Health and Wellbeing to differentiate between groups, analyses were conducted comparing participants by gender (independent-samples t-tests, Welch's correction) and age (one-way ANOVAs across six categories: [18, 30], [31, 40], [41, 50], [51, 60], [61, 70], and >70). Effect sizes were calculated using Cohen's *d* for *t*-tests and inspection of means for ANOVA comparisons. Given the multidimensional structure of Dimension F and the moderate reliability of some sub-domains, results involving this domain should be interpreted with caution.

#### 4.5.1 Gender differences (independent *t*-tests)

- **Dimension A – Self-esteem**. Men (*n* = 138, *M* = 5.15, *SD* = 0.84) scored significantly higher than women (*n* = 5.38, *M* = 4.92, *SD* = 0.92), *t* = 2.89, *p* = 0.004, *d* = 0.26. The effect was small, indicating modest gender differences in self-esteem.
- **Dimension B – Body image (refined)**. Using the revised four-item version of the scale, men (*M* = 5.64, *SD* = 1.04) scored higher than women (*M* = 5.18, *SD* = 1.19). The difference was significant, *t* = 4.59, *p* < 0.001, *d* = 0.40. The effect size was small to moderate, indicating modest gender differences in body-related comfort and perceived stigma.
- **Dimension C – Friendship and social relationships**. No significant gender differences were observed (*Mmen* = 5.69, *Mwomen* = 5.68), *t* = 0.07, *p* = 0.948, *d*≈0.00.
- **Dimension D – Environment**. Differences were not significant (*Mmen* = 5.60, *Mwomen* = 5.83), *t* = −1.52, *p* = 0.130, *d* = −0.16. Women tended to score slightly higher, though the effect was small.
- **Dimension E – Meaningful Work**. No significant differences emerged (*Mmen* = 4.28, *Mwomen* = 4.52), *t* = −1.35, *p* = 0.178, *d* = −0.13. A small, non-significant trend suggested higher female scores.
- **Dimension F – Health Knowledge and Behavior**.

**- Sub-Domain F1 – Preventive Health Behavior**. No significant gender differences were observed. Men (*M* = 4.08, *SD* = 1.52) and women (*M* = 4.25, *SD* = 1.59) reported similar levels of preventive health, behavior *t* = −1.19, *p* = 0.236, *d* = −0.11.
**- Sub-Domain F2 – Attitudes toward Psychological Help**. A significant gender difference was observed. Women (*M* = 6.63, *SD* = 1.12) reported more positive attitudes (i.e., lower stigma) than men (*M* = 6.01, *SD* = 1.39), *t* = −5.05, *p* < 0.001, *d* = −0.52. The effect size was moderate.
**- Sub-Domain F3 – Body-related Perception**. No significant gender differences were observed. Men (*M* = 4.39, *SD* = 1.58) and women (*M* = 4.30, *SD* = 1.64) reported similar levels, *t* = 0.56, *p* = 0.576, *d* = 0.05.
**- Sub-Domain F4 – Dietary Behavior**. A small but significant gender difference was observed. Women (*M* = 3.18, *SD* = 0.92) scored slightly higher than men (*M* = 2.97, *SD* = 0.94), *t* = −2.41, *p* = 0.017, *d* = −0.23.

- **Dimension G – Sense of Future**. No significant differences were found (*Mmen* = 4.54, *Mwomen* = 4.44), *t* = 1.80, *p* = 0.074, *d* = 0.16. A marginal trend suggested slightly higher scores for males.

##### 4.5.1.1 Summary

Gender differences were significant only in *Self-Esteem (A)* and *Body Image (B) [refined]*, with men reporting higher scores. All other dimensions (C–G), as well as the subdomains of Dimension F, did not differ significantly.

#### 4.5.2 Age differences (ANOVAs)

One-way ANOVAs across six age groups revealed the following:

- **Dimension B – Body Image (refined)**. Significant differences were observed, *F*_(5, 671)_ = 10.58, *p* < 0.001. Younger adults (18–30) reported lower body-related comfort and higher perceived body-related stigma, whereas older adults (61–70, 70+) reported higher levels.
- **Dimension C – Friendship and Social Relationships**. Differences across age were significant, *F*_(5, 671)_ = 2.35, *p* = 0.040. Middle-aged participants reported slightly higher levels of social connectedness.
- **Dimension D – Environment**. Significant differences were found, *F*_(5, 671)_ = 2.44, *p* = 0.033, with ecological awareness increasing across age groups, particularly among older adults.
- **Dimension F – Health Knowledge and Behavior**. Given the multidimensional structure of this domain, age-related differences were examined at the sub-domain level. No significant differences were observed for preventive health behavior (F1) or attitudes toward psychological help (F2). However, significant age effects emerged for body-related perception (F3) and dietary behavior (F4), with older participants reporting more health-oriented dietary practices and differences in perceived metabolic functioning.

**- Sub-Domain F1 – Preventive Health Behavior**. No significant age differences were observed, *F*_(5, 671)_ = 1.62, *p* = 0.151, indicating similar levels of preventive health behavior across age groups.
**- Sub-Domain F2 – Attitudes toward Psychological Help**. No significant age differences were found, *F*_(5, 671)_ = 1.34, *p* = 0.245, suggesting that attitudes toward psychological help were relatively stable across age groups.
**- Sub-Domain F3 – Body-related Perception**. Significant differences were observed, *F*_(5, 671)_ = 2.77, *p* = 0.018. This suggests that perceptions related to metabolism and bodily functioning vary across age groups.
**- Sub-Domain F4 – Dietary Behavior**. Significant age differences were observed, *F*_(5, 671)_ = 5.39, *p* < 0.001. Older participants reported more restrictive, health-oriented dietary behaviors than younger participants.

- **Dimension A, E, G – Self-Esteem, Meaningful Work, Present and Future Hope**. No significant age-related differences were observed.

##### 4.5.2.1 Summary

Age effects were evident in body-related, social, environmental, and selected health-related sub-domains (B, C, D, F3, and F4), but not in preventive health behavior (F1), attitudes toward psychological help (F2), self-esteem, work-related perceptions, or hope for the future. These findings suggest that specific aspects of wellbeing, particularly those related to lifestyle and bodily perception, are more sensitive to age than others. However, because measurement invariance across demographic groups was not formally tested in the present study, these group comparisons should be interpreted as preliminary evidence of known-groups validity rather than definitive evidence of structural equivalence across populations.

**Figure 3:**
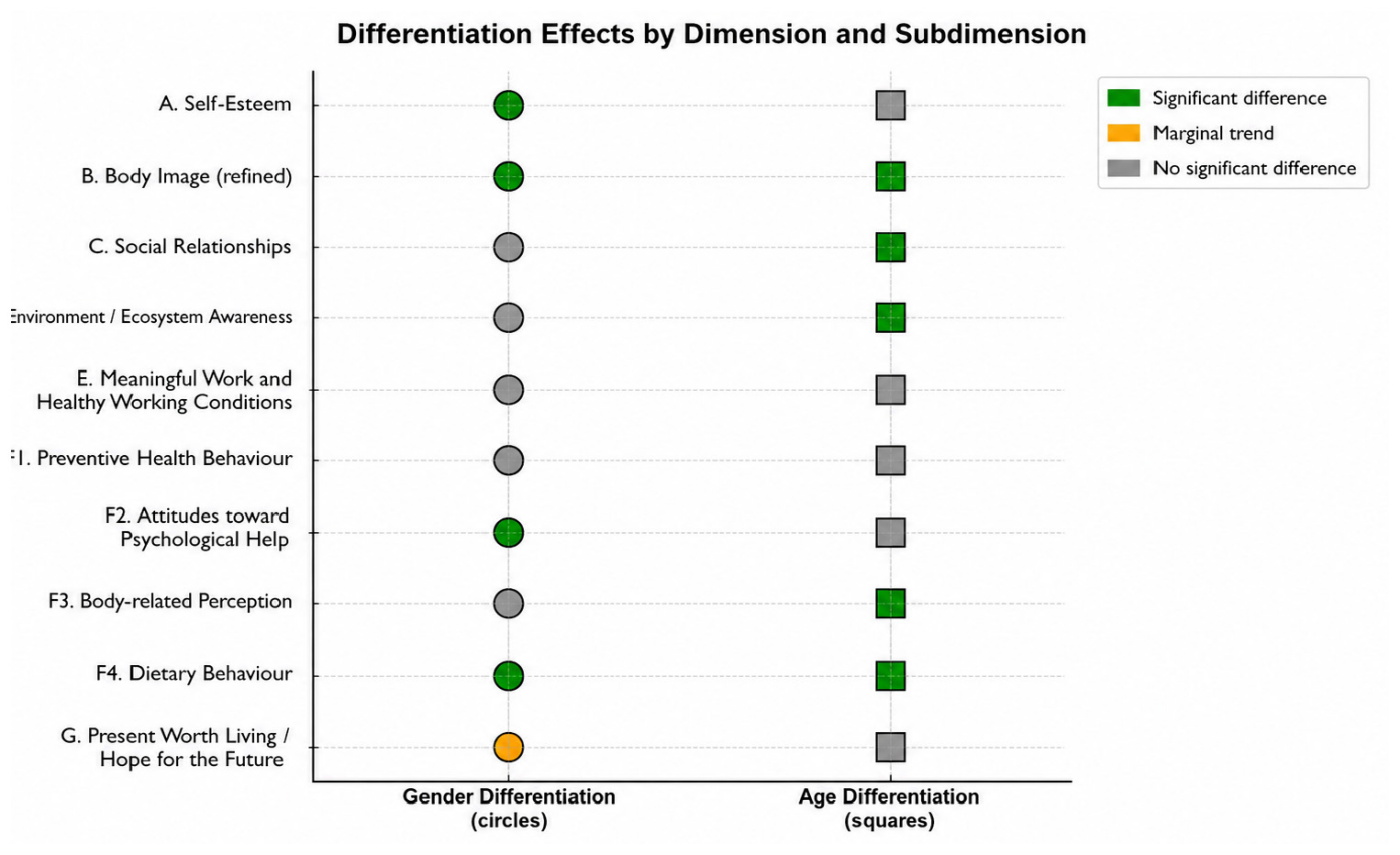
Differentiation effects across dimensions by gender (circles) and age (squares). Green indicates statistically significant differences, yellow indicates marginal trends, and gray indicates no significant differences. The order of dimensions follows the structure of the scale, from A (Self-Esteem, top) to G (Future Hope, bottom).

To visually summarize the differentiation results, Figure 3 presents the effects of gender and age across the dimensions of the Holistic Scale of Body and Mental Health and Wellbeing. Gender differences are displayed as circles, while age-related differences are represented as squares. The color coding indicates the level of significance: green denotes statistically significant differences, orange indicates marginal trends, and gray reflects non-significant effects. As shown, significant gender differences were observed for Self-Esteem (A), Body Image (B, refined), and selected sub-domains of Dimension F, specifically attitudes toward psychological help (F2) and dietary behavior (F4). In contrast, no gender differences were observed for preventive health behavior (F1) or body-related perception (F3).

Age-related effects emerged for Body Image (B, refined), Friendship and Social Relationships (C), Environmental Awareness (D), and selected sub-domains of Dimension F, specifically body-related perception (F3) and dietary behavior (F4), whereas preventive health behavior (F1), attitudes toward psychological help (F2), Self-Esteem (A), Meaningful Work (E), and Future Hope (G) remained stable across age groups. Notably, results for Dimension B are based on the refined four-item version of the scale following reverse coding and removal of conceptually heterogeneous items. For Dimension F, results are presented at the sub-domain level due to its multidimensional structure. This graphical depiction highlights the specific domains where differentiation is most pronounced and those where responses were more consistent across demographic subgroups.

Overall, the differentiation analyses support the scale's construct validity, particularly its known-groups validity. The significant gender effects in Self-Esteem and Body Image are consistent with well-documented patterns in the literature regarding gender differences in self-concept and body-related perceptions. Importantly, gender differences in attitudes toward psychological help (F2) and dietary behavior (F4) further highlight the relevance of examining specific health-related behaviors separately. Similarly, age-related variations in Body Image, Friendship, Environmental Awareness, and selected health-related sub-domains (F3 and F4) align with theoretical expectations that these aspects of wellbeing are shaped by life stage and accumulated experience. The absence of age-related differences in preventive health behavior (F1), attitudes toward psychological help (F2), Self-Esteem, Meaningful Work, and Future Hope highlights the relative stability of these constructs across the lifespan. Together, these results indicate that the instrument is sensitive to group differences where theoretically expected, while also demonstrating stability in more universal aspects of wellbeing. Moreover, the differentiated patterns observed within Dimension F provide further empirical support for its multidimensional structure.

### 4.6 Discussion

The present study examined the psychometric properties and group differentiation of the Holistic Scale of Body and Mental Health and Wellbeing. Beyond reliability and construct validity, we specifically tested whether the instrument could capture meaningful differences across gender and age, as theoretically expected.

#### 4.6.1 Gender differences

The differentiation analysis revealed significant gender differences in Self-Esteem and Body Image (refined), with men reporting higher levels in both dimensions. These findings are consistent with a large body of research documenting persistent gender differences in body image and self-concept. Women tend to report greater body dissatisfaction and lower self-esteem, partially explained by sociocultural pressures, internalized appearance norms, and differential exposure to body-related stigma (Tiggemann, 2004; Frederick and Essayli, 2016). The small-to-moderate effect size for Body Image compared to the smaller effect for Self-Esteem is in line with meta-analytic evidence showing stronger gender gaps in body-related constructs than in general self-esteem (Orth and Robins, 2014).

Importantly, additional gender differences emerged within specific sub-domains of Dimension F. Women reported significantly more positive attitudes toward psychological help (F2), reflecting lower levels of stigma, as well as slightly higher engagement in health-oriented dietary behaviors (F4). These findings align with prior literature indicating that women are generally more open to seeking psychological support and tend to adopt more health-conscious lifestyle behaviors (Mackenzie et al., 2006; Wardle et al., 2004). In contrast, no gender differences were observed in preventive health behavior (F1) or body-related perception (F3), suggesting that these aspects of health-related functioning may be less influenced by gender.

No significant gender differences were found in Friendship and Social Relationships, Environment, Meaningful Work, or Future Hope. This suggests that while gender differences are pronounced in domains directly linked to self-concept, body evaluation, and certain health behaviors, they are less salient in broader psychosocial, ecological, or existential dimensions of wellbeing. Small non-significant trends were nevertheless observed, such as women scoring slightly higher in environmental awareness and meaningful work, and men reporting marginally higher levels of hope for the future. These findings align with prior evidence indicating that gender gaps in environmental attitudes and work perceptions are context-dependent and less consistent than those related to body image (Zelezny et al., 2000; Schieman and Glavin, 2011).

#### 4.6.2 Age differences

The ANOVAs revealed significant age-related variation in Body Image (refined), Friendship and Social Relationships, Environmental Awareness, and selected sub-domains of Dimension F. As expected, body-related comfort increased with age, reflecting the well-established finding that older adults tend to show greater body acceptance and less appearance-related anxiety compared to younger cohorts (Tiggemann, 2004). Middle-aged participants reported comparatively higher levels of social connectedness, consistent with developmental theories that emphasize the consolidation of social networks and community integration during midlife (Carstensen, 1992). Environmental awareness also increased across age groups, in line with evidence that ecological concern is reinforced by life experience and greater awareness of long-term environmental risks (Dietz, 2007).

Within Dimension F, age differences were not uniform across sub-domains. No significant variation was observed in preventive health behavior (F1) or attitudes toward psychological help (F2), suggesting that these behaviors remain relatively stable across adulthood. However, significant differences emerged in body-related perception (F3) and dietary behavior (F4). Older participants reported more health-oriented dietary practices, consistent with previous research showing higher levels of dietary regulation and health-conscious behavior among older adults (Wardle et al., 2004). Differences in body-related perceptions may reflect age-related changes in physiological awareness and in the interpretation of bodily functioning (Stephan et al., 2013).

In contrast, no significant differences were found across age groups for Self-Esteem, Meaningful Work, or Future Hope. These results suggest that such constructs may remain relatively stable across the adult lifespan. Prior longitudinal studies have similarly shown that self-esteem follows a relatively stable trajectory in adulthood (Orth and Robins, 2014), while meaning in work and existential hope are influenced more by contextual and cultural factors than by age alone (Steger et al., 2012).

#### 4.6.3 Implications of the model evaluation and future refinements of non-reliable dimensions

Together, these findings support the scale's construct validity, specifically its known-groups validity. The instrument successfully differentiated between groups in areas where theoretical and empirical work predict differences, including gender effects on self- and body-related constructs and age-related differences across body, social, environmental, and selected health-related domains. At the same time, the stability of certain constructs (self-esteem, meaningful work, and hope for the future) across demographic groups suggests that the scale is not overly sensitive to spurious variance. This pattern supports the conceptual coherence of the instrument, as it captures differentiation where theoretically expected, while preserving stability in more general dimensions of wellbeing.

The present study provides an empirical examination of the Holistic Scale of Body and Mental Health and Wellbeing within a German-speaking population, enabling us to evaluate how the 7DHW theoretical model's seven dimensions are operationalized in this cultural context. The model's dimensional structure, grounded in the WHO definition of health and organized around Self-Esteem, Body Image, Social Relationships, Environment, Meaningful Work, Health Knowledge and Behavior, and Sense of Future, was supported by the instrument's psychometric properties. Five of the seven dimensions (A, C, D, E, and G) demonstrated good to excellent internal consistency, suggesting robust coherence among their items and strong construct representation.

Dimension B (Body Image) initially showed very poor reliability. However, item-level diagnostics identified a polarity issue and conceptual heterogeneity within the original item set. After reverse coding the body-shaming items and removing two conceptually misaligned indicators, the refined four-item version demonstrated substantially improved internal consistency and conceptual coherence. This suggests that the construct is measurable within the proposed framework.

In contrast, Dimension F (Health Knowledge and Behavior) did not function as a unidimensional construct. Both exploratory and confirmatory analyses indicated a heterogeneous structure, with distinct components related to preventive behavior, dietary practices, attitudes toward psychological help, and body-related perception. Rather than reflecting a measurement failure, these findings suggest that Dimension F captures a multidimensional domain comprising partially independent subcomponents. Subsequent analyses at the sub-domain level demonstrated more interpretable reliability and meaningful differentiation patterns across gender and age, supporting this interpretation. These results indicate that Dimension F may be more appropriately conceptualized and modeled as a set of related sub-domains rather than a single composite scale.

Overall, these findings support the interpretation of the present study as a validation-and-refinement effort of the full instrument. The results reinforce the potential of the 7DHW framework while highlighting the importance of iterative psychometric development. The variability observed does not undermine the theoretical model.

Accordingly, interpretation of the instrument is currently recommended at the domain and sub-domain level rather than through a single aggregated 7DHW total score.

#### 4.6.4 Limitations and future directions

Despite these encouraging results, several limitations must be noted. The sample was unevenly distributed by gender, with a higher proportion of women than men, which may have influenced the precision of the effect size estimates. Additionally, although significant differences were detected across age groups, the cross-sectional design precludes strong conclusions about developmental trajectories. Future research should incorporate longitudinal designs to examine within-individual changes over time.

The recruitment context may also have influenced specific dimensions of the instrument. Because participants were recruited from a rehabilitation and physiotherapy service database, the sample may have been enriched for individuals with greater health awareness, prior engagement with healthcare services, and increased interest in wellbeing-related topics. This may have particularly influenced responses in domains such as body image, preventive health behaviors, dietary practices, physical functioning, and attitudes toward psychological help-seeking. Consequently, the observed factor structures and group differences may not fully generalize to the broader German adult population, particularly to younger individuals or those without prior rehabilitation or healthcare-related experiences.

The sample was not population-based but instead consisted of a convenience sample drawn from a database of former clients of a rehabilitation and physiotherapy service, which may limit generalizability. This sampling strategy, combined with the observed gender imbalance and the relatively high mean age, limits the generalizability of the findings. The results should therefore be interpreted as reflecting a German adult convenience sample.

A further methodological limitation concerns the internal consistency and structure of certain dimensions. In its initial form, Dimension B (Body Image) demonstrated very low reliability (α = 0.012, ω = 0.360), primarily due to misaligned item polarity and conceptual heterogeneity. Following item-level diagnostics, reverse coding of negatively worded items and removal of conceptually misaligned indicators led to a refined four-item version with substantially improved internal consistency (α = 0.756, ω = 0.758). While this revision enhances the construct's interpretability in the present study, further validation in independent samples is required to confirm its stability and generalizability.

Dimension F (Health Knowledge and Behavior) also presented methodological challenges, not solely due to low reliability, but also because of its heterogeneous structure. Exploratory and confirmatory analyses indicated that this dimension comprises multiple partially independent subcomponents, including preventive health behavior, dietary practices, attitudes toward psychological help, and body-related perception. Subsequent analyses conducted at the sub-domain level yielded more interpretable results, supporting a multidimensional conceptualization of this domain.

Finally, the present study did not examine test–retest reliability, measurement invariance across demographic groups, or convergent, discriminant, and criterion-related validity with established instruments. These forms of validity evidence will be essential before the questionnaire can be recommended for broader research or applied use. In particular, measurement invariance testing across gender, age, and other demographic groups will be necessary to determine whether observed group differences reflect true variation in wellbeing constructs rather than differences in item interpretation or measurement functioning. Similarly, convergent and discriminant validity analyses using established wellbeing, health literacy, body image, and psychological functioning instruments will be important to further clarify the distinctiveness and external validity of the proposed domains.

Future studies should therefore evaluate the instrument in more demographically diverse and population-based samples to determine the stability and generalizability of the observed psychometric structure across different age groups, health backgrounds, and cultural contexts.

## 5 Conclusion

The present study provides a psychometric evaluation of the Holistic Scale of Body and Mental Health and Wellbeing, a multidimensional instrument designed to capture the psychological, social, and environmental determinants of wellbeing. The findings support the conceptual relevance of the seven-domain framework, while indicating that several domains are inherently multidimensional and cannot be adequately represented as strictly unidimensional scales.

The scale demonstrated promising psychometric performance across several dimensions, particularly Self-Esteem, Social Relationships, Environment, Meaningful Work, and Sense of the Future. In addition, the instrument differentiated between groups in theoretically meaningful ways. Gender differences were observed in Self-Esteem and Body Image (refined), as well as in specific health-related sub-domains, namely attitudes toward psychological help and dietary behavior. Age-related differences emerged in Body Image, Social Relationships, Environmental Awareness, and selected health-related sub-domains, particularly dietary behavior and body-related perception, while other constructs remained relatively stable across groups.

Importantly, the findings also identified areas requiring further development. Dimension B (Body Image) initially showed poor psychometric performance but demonstrated improved internal consistency following item refinement and reverse coding. Nevertheless, the factorial structure of the refined version remains provisional and should be confirmed in independent samples. In contrast, Dimension F (Health Knowledge and Behavior) did not function as a unidimensional construct and was better represented as a set of exploratory sub-domains with varying levels of reliability. These findings highlight the importance of modeling health-related behaviors and attitudes as heterogeneous components rather than a single aggregate dimension.

Taken together, these results position the present study as a domain-level validation and refinement of the instrument. While several dimensions show satisfactory psychometric properties, others require further development and confirmation in independent samples. Future research should focus on refining weaker dimensions, testing revised versions in more diverse populations, and evaluating alternative structural models, including multidimensional and hierarchical approaches. Such efforts will be essential to fully establish the scale's psychometric robustness and practical applicability in both research and applied settings.

## Data Availability

The datasets presented in this article are not readily available because the dataset contains anonymized participant responses; however, it is not publicly available due to data protection and confidentiality considerations. Although no personally identifiable information was collected, the data originate from a defined participant pool and are subject to institutional and GDPR regulations. Access to the dataset may be granted by the corresponding author upon reasonable request, provided that the intended use complies with ethical standards and data protection requirements. Requests to access the datasets should be directed to Inês Santos Silva, ines.santossilva@2256c.health.
